# 1919. A Year's Work in the Hospitals and Medical Charities of London

**Published:** 1920-06-26

**Authors:** 


### 1919.

### A YEAR'S WORK IN THE HOSPITALS AND MEDICAL CHARITIES OF LONDON.

### ST. MARYLEBONE AND WEST CENTRAL DISTRICT.

Comprising St. Marylebone, St. John's Wood, Bloomsbury, Holborn, &c.

| No. of Beds. | No. of Beds Daily Occupied. | HOSPITALS. | In-patients. | Out-patient Attendances. | Total Expenditure. | Income. | | | Total Income. | Legacies not included in preceding column |
|---|---|---|---|---|---|---|---|---|---|---|
| | | | | | | Charitable. | Proprietary. | Patients' Payments. | | |
| | | | | | £ | £ | £ | £ | £ | £ |
| 74 | 46 | French ... ... ... ... | 854 | 19,291 | 8,720 | 6,152 | 2,623 | 128 | 8,903 | 902 |
| 50 | 36 | Italian ... ... ... ... | 757 | 5,517 | 8,541 | 2,688 | 2,318 | 804 | 5,810 | ... |
| 162 | 109 | London Homœopathic ... ... | 1,507 | 52,209 | 24,333 | 9,680 | 4,291 | 2,459 | 16,430 | 190 |
| 122 | 105 | SS. John and Elizabeth ... ... | 1,118 | ... | 20,182 | 2,613 | 2,815 | 4,731 | 10,159 | 16,443 |
| 361 | 192 | The Middlesex ... ... ... | 3,325 | 121,679 | 85,612 | 69,582 | 12,133 | 2,616 | 81,331 | 10,676 |
| 68 | 64 | Alexandra, for Children... ... | 65 | 386 | 8,209 | 3,735 | 1,348 | 5,209 | 10,292 | 1,050 |
| 223 | 200 | Hospital for Sick Children ... | 2,727 | 102,260 | 37,374 | 15,614 | 7,531 | 3,384 | 26,529 | 2,620 |
| 24 | 20 | S. Monica's, for Children ... | 95 | ... | 2,004 | 861 | 151 | 525 | 1,537 | ... |
| 71 | 67 | Queen Charlotte's Lying-In ... | 1,991 | 19,530 | 15,888 | 9,185 | 1,306 | 3,120 | 13,611 | 135 |
| 71 | 67 | Elizabeth Garrett Anderson Hospital ... ... ... | 1,365 | 41,128 | 17,806 | 6,694 | 2,760 | 4,708 | 14,162 | 1,985 |
| 70 | 58 | Samaritan Free ... ... ... | 1,068 | 14,752 | 11,795 | 6,452 | 1,323 | ... | 7,775 | 1,470 |
| 199 | 177 | National for the Paralysed, &c.... | 963 | 53,086 | 38,752 | 10,293 | 2 996 | 14,096 | 27,385 | 4,472 |
| 85 | 83 | Hospital for Epilepsy, &c. ... | 497 | 22,709 | 10,802 | 5,724 | 681 | 3,336 | 9,741 | 500 |
| 102 | 69 | West End, for Epilepsy, &c. ... | 438 | 37,826 | 13,324 | 5,492 | 1,727 | 4,725 | 11,944 | 25 |
| 40 | 27 | Central London Ophthalmic ... | 488 | 29,383 | 4,870 | 2,944 | 642 | 61 | 3,647 | 5,726 |
| 16 | 15 | Western Ophthalmic ... ... | 363 | 23,854 | 3,339 | 2,822 | 279 | 597 | 3,698 | ... |
| 191 | 170 | Royal National Orthopædic ... | 1,121 | 59,181 | 26,390 | 6,796 | 1,795 | 14,216 | 22,807 | 1,950 |
| 38 | 31 | Florence Nightingale Home ... | 460 | ... | 6,971 | 2,163 | 551 | 3,045 | 5,759 | 750 |
| 92 | 78 | The Middlesex Cancer ... ... | 198 | 513 | 21,520 | 3,765 | 10,666 | ... | 14,431 | 3,020 |
| 2,059 | 1,614 | | 19,400 | 603,304 | 366,432 | 173,255 | 57,936 | 67,760 | 298,951 | 51,914 |
| | | DISPENSARIES. | | | | | | | | |
| ... | ... | London Medical Mission ... | ... | 1,948 | 1,067 | 420 | 278 | 214 | 912 | ... |
| ... | ... | Margaret Street, for Consumption | ... | 6,941 | 1,068 | 439 | 711 | ... | 1,150 | ... |
| ... | ... | St. John's Wood Provident ... | ... | 21,000 | 505 | 362 | 27 | 158 | 547 | ... |
| ... | ... | St. Marylebone General ... ... | ... | 14,823 | 1,946 | 581 | 289 | 462 | 1,332 | ... |
| ... | ... | Western General ... ... ... | ... | 11,070 | 967 | 531 | 202 | 17 | 750 | 300 |
| 2,059 | 1,614 | | 19,400 | 659,086 | 371,985 | 175,588 | 59,443 | 68,611 | 303,642 | 52,214 |

### WESTMINSTER DISTRICT.—Comprising Westminster City and Liberties.

| No. of Beds. | No. of Beds Daily Occupied. | HOSPITALS. | In-patients. | Out-patient Attendances. | Total Expenditure. | Charitable. | Proprietary. | Patients' Payments. | Total Income. | Legacies not included in preceding column |
|---|---|---|---|---|---|---|---|---|---|---|
| | | | | | £ | £ | £ | £ | £ | £ |
| 238 | 183 | Charing Cross ... ... ... | 2,757 | 90,126 | 53,032 | 50,079 | 4,904 | 3,500 | 58,483 | 11,927 |
| 213 | 176 | Westminster ... ... ... | 2,107 | 93,094 | 39,159 | 11,827 | 5,516 | 2,859 | 20,202 | 3,683 |
| 177 | 167 | Ventnor, for Consumption ... | 719 | ... | 24,646 | 6,688 | 3,747 | 11,420 | 21,855 | 172 |
| 37 | 22 | Grosvenor, for Women & Children | 347 | 6,458 | 4,239 | 2,071 | 653 | 802 | 3,526 | ... |
| 68 | 65 | Hospital for Women ... ... | 1,073 | 14,056 | 10,736 | 5,206 | 901 | 2,234 | 8,341 | 225 |
| 42 | 38 | National, for Diseases of Heart... | 291 | 28,806 | 8,285 | 2,773 | 1,136 | 3,034 | 6,943 | 834 |
| 42 | 30 | Male Lock ... ... ... | 475 | 62,575 | 8,966 | 1,540 | 5 | 6,446 | 7,991 | 1,075 |
| 40 | 29 | Royal Westminster Ophthalmic... | 676 | 33,837 | 5,648 | 2,661 | 1,534 | 145 | 4,340 | ... |
| ... | ... | Royal Dental ... ... ... | ... | 31,573 | 5,876 | 3,767 | 2,181 | 37 | 5,985 | 105 |
| 32 | 25 | St. Peter's (Stone) ... ... | 430 | 31,538 | 8,256 | 2,197 | 1,046 | 2,758 | 6,001 | 1,200 |
| 50 | 35 | Infants' Hospital, Vincent Square | 266 | 2,837 | 5,325 | 3,776 | 79 | ... | 3,855 | 450 |
| 28 | 19 | St. John's, for Skin Diseases ... | 162 | 45,812 | 6,683 | 2,102 | 118 | 4,079 | 6,299 | 250 |
| 967 | 789 | | 9,303 | 440,712 | 180,851 | 94,687 | 21,820 | 37,314 | 153,821 | 19,921 |
| | | DISPENSARIES. | | | | | | | | |
| ... | ... | Public ... ... ... ... | ... | 9,189 | 729 | 413 | 194 | ... | 607 | 72 |
| ... | ... | St. George's, Hanover Square ... | ... | 1,393 | 402 | 227 | 89 | 69 | 385 | ... |
| ... | ... | Western ... ... ... ... | ... | 8,737 | 1,664 | 303 | 510 | 571 | 1,384 | ... |
| ... | ... | Westminster General ... ... | ... | 11,300 | 1,265 | 312 | 507 | 103 | 922 | ... |
| 967 | 789 | | 9,303 | 471,331 | 184,911 | 95,942 | 23,120 | 38,057 | 157,119 | 19,993 |

### CITY AND EAST CENTRAL DISTRICT.

**Comprising the City, St. Luke's, Shoreditch, Finsbury, and Clerkenwell.**

| No. of Beds. | No. of Beds Daily Occupied. | HOSPITALS. | In-patients. | Out-patient Attendances. | Total Expenditure. | Income. | | | Total Income. | Legacies not included in preceding column. |
|---|---|---|---|---|---|---|---|---|---|---|
| | | | | | | Charitable. | Proprietary. | Patients' Payments. | | |
| | | | | | £ | £ | £ | £ | £ | £ |
| 405 | 167 | Metropolitan ... ... ... | 2,381 | 161,930 | 35,757 | 15,469 | 1,183 | 12,468 | 29,120 | 2,523 |
| 204 | 158 | Royal Free ... ... ... | 2,875 | 148,441 | 47,189 | 13,367 | 5,933 | 3,767 | 23,067 | 3,750 |
| 686 | 494 | St. Bartholomew's ... ... | 7,729 | 271,289 | 150,969 | 55,782 | 78,415 | 9,776 | 143,973 | 146 |
| 80 | 48 | Royal, for Diseases of the Chest. | 468 | 33,487 | 14,697 | 6,573 | 592 | 6,278 | 13,443 | 162 |
| 148 | 141 | Queen's, for Children ... ... | 1,615 | 108,306 | 29,887 | 21,439 | 1,380 | 1,127 | 23,946 | 1,120 |
| 71 | 58 | City of London Maternity ... | 1,486 | 6,484 | 12,412 | 6,258 | 3,757 | 2,543 | 12,558 | ... |
| 11 | 10 | Jewish Maternity Hospital ... | 293 | 494 | 2,924 | 1,513 | 445 | 909 | 2,867 | 140 |
| 56 | 41 | St. Mark's, for Fistula ... ... | 655 | 4,259 | 9,381 | 7,502 | 1,566 | 351 | 9,419 | 60 |
| 138 | 109 | Royal London Ophthalmic ... | 2,557 | 105,526 | 24,936 | 10,514 | 2,259 | 1,584 | 14,357 | 3,242 |
| 41 | 33 | Central London Throat and Ear. | 824 | 48,163 | 7,917 | 2,168 | 496 | 5,540 | 8,204 | 650 |
| 1,840 | 1,259 | | 20,883 | 888,379 | 336,069 | 140,585 | 96,026 | 44,343 | 280,954 | 11,793 |
| | | DISPENSARIES. | | | | | | | | |
| ... | ... | Billingsgate Medical Mission ... | ... | 11,630 | 785 | 555 | ... | 132 | 687 | ... |
| ... | ... | City ... ... ... ... | ... | 11,465 | 903 | 766 | 117 | ... | 883 | ... |
| ... | ... | Farringdon General ... ... | ... | 4,820 | 753 | 393 | 14 | 178 | 585 | ... |
| ... | ... | Finsbury ... ... ... ... | ... | 26,448 | 1,359 | 430 | 187 | 327 | 944 | 450 |
| ... | ... | Metropolitan ... ... ... | ... | 2,518 | 676 | 207 | 382 | 94 | 683 | ... |
| ... | ... | Royal General ... ... ... | ... | 5,175 | 850 | 218 | 388 | 43 | 649 | ... |
| 1,840 | 1,259 | | 20,883 | 950,435 | 341,395 | 143,154 | 97,114 | 45,117 | 285,385 | 12,243 |

### ISLINGTON AND NORTH-WEST DISTRICT.

**Comprising Islington, Holloway, Highbury, Hampstead, Highgate, St. Pancras, Stoke Newington, Tottenham, &c.**

| No. of Beds. | No. of Beds Daily Occupied. | HOSPITALS. | In-patients. | Out-patient Attendances. | Total Expenditure. | Income. | | | Total Income. | Legacies not included in preceding column. |
|---|---|---|---|---|---|---|---|---|---|---|
| | | | | | | Charitable. | Proprietary. | Patients' Payments. | | |
| | | | | | £ | £ | £ | £ | £ | £ |
| 232 | 207 | Great Northern Central ... ... | 3,044 | 173,637 | 58,277 | 34,372 | 2,117 | 15,498 | 51,987 | 1,031 |
| 128 | 112 | Hampstead General Hospital ... | 1,414 | 28,676 | 20,456 | 16,089 | 1,497 | 2,965 | 20,551 | 1,300 |
| 105 | 85 | London Temperance ... ... | 1,223 | 82,897 | 15,216 | 7,014 | 2,245 | 1,478 | 10,737 | 851 |
| 125 | 101 | Tottenham (Prince of Wales's)... | 1,893 | 84,573 | 18,155 | 15,263 | 1,249 | 1,465 | 17,977 | 110 |
| 322 | 309 | University College ... ... | 3,810 | 183,978 | 57,482 | 21,266 | 7,194 | 14,952 | 43,412 | 2,343 |
| 110 | 100 | Mount Vernon, for Consumption | 343 | 13,970 | 20,273 | 3,154 | 4,349 | 7,759 | 15,262 | 157 |
| 20 | 19 | Children's Home Hospital, Barnet | 31 | ... | 866 | 412 | 335 | 55 | 802 | ... |
| 178 | 60 | London Fever ... ... ... | 827 | ... | 18,457 | 6,429 | 1,909 | 6,142 | 14,480 | 708 |
| 72 | 71 | Salvation Army Mothers' ... | 1,078 | ... | 9,553 | 1,397 | 2,195 | 6,135 | 9,727 | 20 |
| 25 | 20 | Enfield Cottage ... ... ... | 296 | ... | 2,109 | 1,205 | 117 | 263 | 1,585 | ... |
| 23 | 16 | Hendon Cottage ... ... ... | 299 | 1,903 | 3,421 | 1,208 | 109 | 1,236 | 2,553 | ... |
| 21 | 15 | St. Saviour's Hospital ... ... | 141 | ... | 3,122 | 1,604 | 295 | 1,269 | 3,168 | ... |
| 48 | 23 | St. Columba's Hospital ... ... | 116 | ... | 7,413 | 6,124 | 443 | 380 | 6,947 | 50 |
| 72 | 31 | Willesden Cottage ... ... | 398 | ... | 5,771 | 1,975 | 407 | 1,916 | 4,298 | 500 |
| 20 | 20 | Santa Claus Home ... ... | 22 | ... | 1,635 | 1,325 | 203 | 103 | 1,631 | ... |
| 25 | 12 | Stoke Newington Home Hospital | 150 | ... | 1,796 | 895 | 406 | 418 | 1,719 | ... |
| 18 | 17 | Winifred House, Holloway ... | 26 | ... | 1,396 | 490 | 196 | 283 | 969 | 81 |
| 21 | 1 | Highgate Convalescent Home ... | 140 | ... | 769 | 524 | 41 | 177 | 742 | ... |
| 50 | 34 | St. Andrew's, Dollis Hill ... | 660 | ... | 7,514 | 1,995 | 3,839 | 714 | 6,548 | ... |
| 1,615 | 1,273 | | 15,911 | 569,634 | 253,681 | 122,741 | 29,146 | 63,208 | 215,095 | 7,151 |
| | | DISPENSARIES | | | | | | | | |
| ... | ... | Child's Hill Provident ... ... | ... | 3,900 | 135 | 17 | 8 | 88 | 113 | ... |
| ... | ... | Hampstead Provident ... ... | ... | 10,888 | 897 | 307 | 54 | 546 | 907 | ... |
| ... | ... | Islington ... ... ... | ... | 42,696 | 1,007 | 207 | 111 | 743 | 1,061 | ... |
| ... | ... | Islington Medical Mission ... | ... | 15,817 | 1,265 | 523 | 428 | 158 | 1,109 | ... |
| ... | ... | St. Pancras and Northern ... | ... | 7,179 | 1,822 | 310 | 153 | 1,569 | 2,032 | ... |
| ... | ... | Stamford Hill, &c. ... ... | ... | 15,356 | 731 | 605 | 156 | ... | 761 | ... |
| 1,6 5 | 1,273 | | 15,911 | 665,470 | 259,538 | 124,710 | 30,056 | 66,312 | 221,078 | 7,151 |

### STRATFORD AND EAST-END DISTRICT.

**Comprising Bethnal Green, Tower Hamlets, West Ham, Whitechapel, Hackney, Stepney, Limehouse, Poplar, and the East.**

| No. of Beds. | No. of Beds Daily Occupied. | Hospitals. | In-patients | Out-patient Attendances. | Total Expenditure. | Income. Charitable. | Income. Proprietary. | Income. Patients' Payments. | Total Income. | Legacies not included in preceding column. |
|---|---|---|---|---|---|---|---|---|---|---|
| | | | | | £ | £ | £ | £ | £ | £ |
| 933 | 774 | London ... ... ... ... | 18,962 | 450,136 | 239,873 | 95,256 | 36,948 | 14,663 | 146,867 | 12,620 |
| 52 | 37 | Mildmay Mission Hospital ... | 541 | 27,009 | 6,754 | 4,771 | 659 | 678 | 6,108 | ... |
| 29 | 15 | Mildmay Memorial ... ... | 208 | ... | 2,961 | 1,562 | 1,167 | 436 | 3,165 | 36 |
| 115 | 79 | Poplar ... ... ... ... | 1,871 | 77,770 | 17,324 | 20,440 | 7,844 | 474 | 28,758 | 20 |
| 50 | 39 | Walthamstow, &c. ... ... | 523 | 15,208 | 3,718 | 2,863 | 667 | 36 | 3,566 | 200 |
| 130 | 110 | Queen Mary's, &c. ... ... | 1,752 | 144,919 | 27,293 | 19,251 | 2,102 | 3.526 | 24,879 | 475 |
| 175 | 163 | City of London for Dis. of the Chest | 889 | 33,624 | 29,674 | 17,654 | 1,858 | 9,409 | 28,921 | 567 |
| 130 | 83 | East London, for Children ... | 1,081 | 61,777 | 18,796 | 13,766 | 3,740 | 200 | 17,706 | 2,362 |
| 43 | 42 | St. Mary's, Plaistow, for Children | 716 | 28,760 | 6,920 | 4,418 | 1,040 | 192 | 5,650 | ... |
| 33 | 29 | East End Mothers' Home ... | 801 | 19,934 | 4,681 | 2,317 | 1,560 | 917 | 4,794 | 20 |
| 23 | 15 | Plaistow Maternity ... ... | 476 | ... | 1,979 | 1,306 | 21 | 464 | 1,791 | ... |
| 12 | 9 | Brentwood Cottage ... ... | 158 | — | 1,273 | 520 | 95 | 294 | 909 | ... |
| 26 | 18 | Canning Town Cottage ... ... | 266 | 8,398 | 3,341 | 2,181 | 317 | 576 | 3,074 | 100 |
| 17 | 10 | Passmore Edwards Cottage, T'lb'ry | 201 | 2,647 | 997 | 1,371 | 101 | 22 | 1,494 | ... |
| 25 | 10 | East Ham Cottage ... ... | 119 | 14,369 | 1,617 | 816 | 524 | 154 | 1,494 | — |
| 154 | 102 | German ... ... ... ... | 1,718 | 42,221 | 14,046 | 3,641 | 5,484 | 1,346 | 10,471 | 482 |
| 1,947 | 1,535 | | 30,282 | 926,772 | 381,247 | 192,133 | 64,127 | 33,387 | 289,647 | 16,882 |
| | | **Dispensaries.** | | | | | | | | |
| ... | ... | Buxton Street ... ... ... | ... | 1,187 | 113 | 90 | ... | ... | 90 | ... |
| — | ... | Eastern ... ... ... ... | ... | 20,179 | 925 | 199 | 248 | 432 | 879 | ... |
| — | ... | London ... ... ... ... | ... | 2,528 | 494 | 85 | 277 | ... | 362 | ... |
| ... | ... | Queen Adelaide's ... ... ... | ... | 9,171 | 700 | 432 | 632 | ... | 1,064 | ... |
| 1,947 | 1,535 | | 30,282 | 959,837 | 383,479 | 192,939 | 65,284 | 33,819 | 292,042 | 16,882 |

### KENSINGTON AND WEST DISTRICT.

**Comprising Kensington, Paddington, Bayswater, Kilburn, Chelsea, Brompton, Fulham, Hammersmith, Chiswick, Brentford, Acton, Ealing, &c.**

| No. of Beds. | No. of Beds Daily Occupied. | Hospitals. | In-patients | Out-patient Attendances. | Total Expenditure. | Income. Charitable. | Income. Proprietary. | Income. Patients' Payments. | Total Income. | Legacies not included in preceding column. |
|---|---|---|---|---|---|---|---|---|---|---|
| | | | | | £ | £ | £ | £ | £ | £ |
| 336 | 302 | St. George's ... ... ... | 4,685 | 147,011 | 69,201 | 16,948 | 13,754 | 5,107 | 35,809 | 11,820 |
| 274 | 234 | St. Mary's ... ... ... ... | 3,591 | 121,057 | 55,126 | 17,597 | 10,985 | 6,473 | 35,055 | 24,937 |
| 144 | 135 | West London ... ... ... | 1,945 | 174,625 | 37,141 | 17,938 | 2,726 | 2,826 | 23,490 | 953 |
| 481 | 435 | Hospital for Consumption ... | 1,845 | 29,649 | 71,270 | 22,598 | 4,376 | 40,796 | 67,770 | 6,542 |
| 50 | 49 | Cheyne, for Sick & Incurable Chldn | 45 | ... | 7,201 | 2,597 | 1,612 | 1,894 | 6,103 | 1,150 |
| 22 | 19 | Kensington, General ... ... | 200 | 32,543 | 3,853 | 2,161 | 904 | 91 | 3,156 | 93 |
| 11 | 5 | Kensington, for Children .. | 158 | 8,374 | 1,470 | 946 | 220 | ... | 1,166 | ... |
| 46 | 33 | Paddington Green, for Children | 760 | 41,300 | 9,993 | 4,387 | 2,361 | 490 | 7,238 | 26,539 |
| 104 | 75 | Victoria, for Children ... ... | 1,041 | 52,940 | 15,811 | 8,060 | 2,768 | 693 | 11,521 | 625 |
| 50 | 43 | Chelsea, for Women ... ... | 760 | 7,788 | 10,038 | 8,336 | 1,476 | 1,890 | 11,702 | 2,053 |
| 127 | 82 | Cancer ... ... ... ... | 631 | 25,728 | 29,387 | 4,420 | 10,515 | ... | 14,935 | 20,199 |
| 162 | 139 | Female Lock ... ... ... | 636 | ... | 14,184 | 6,080 | 1,010 | 7,014 | 14,104 | 2,152 |
| 24 | 22 | Banstead Surgical Home ... | 60 | ... | 1,390 | 306 | 75 | 587 | 968 | 250 |
| 35 | 28 | Acton Cottage ... ... ... | 335 | 11,814 | 3,102 | 2,452 | 280 | 384 | 3,119 | 100 |
| 50 | 37 | Ealing Cottage ... ... ... | 627 | ... | 6,347 | 2,428 | 1,221 | 1,326 | 4,975 | 963 |
| 18 | 12 | Epsom and Ewell Cottage ... | 291 | ... | 1,902 | 1,154 | 134 | 629 | 1,917 | ... |
| 27 | 20 | Hounslow Cottage ... ... | 247 | 7,881 | 3,391 | 1,131 | 402 | 1,006 | 2,539 | ... |
| 52 | 41 | Reigate and Redhill Cottage ... | 731 | ... | 4,798 | 3,993 | 541 | 773 | 5,307 | 550 |
| 21 | 16 | Surbiton Cottage ... ... | 301 | ... | 1,767 | 700 | 599 | 327 | 1,626 | ... |
| 32 | 25 | Wimbledon ... ... ... | 428 | 116 | 3,057 | 1,963 | 658 | 746 | 3,367 | 600 |
| 35 | 30 | Wimbledon (Nelson) ... ... | 507 | 3,949 | 3,526 | 2,388 | 301 | 1,336 | 4,025 | 150 |
| 30 | 26 | St. Luke's ... ... ... | 171 | ... | 3,223 | 1,839 | 560 | 833 | 3,232 | 100 |
| 2,131 | 1,808 | | 19,995 | 664,775 | 357,178 | 130,422 | 57,481 | 75,221 | 263,124 | 99,776 |
| | | **Dispensaries.** | | | | | | | | |
| — | — | Kilburn, Maida Vale ... ... | ... | 5,071 | 342 | 242 | 39 | ... | 281 | ... |
| — | — | Kilburn Provident ... ... | ... | 9,893 | 1,061 | 79 | 20 | 980 | 1,079 | ... |
| — | — | Notting Hill Provident ... ... | ... | 8,611 | 439 | 74 | 26 | 351 | 451 | ... |
| 2,131 | 1,808 | | 19,995 | 688,350 | 359,020 | 130,817 | 57,566 | 76,552 | 264,935 | 99,776 |

### NEWINGTON AND SOUTH DISTRICT.

Comprising Battersea, Wandsworth, Tooting, Balham, Streatham, Brixton, Lambeth, Newington, Southwark, Bermondsey, Camberwell, Greenwich, Deptford, Lewisham, Blackheath, Woolwich, &c.

| No. of Beds. | No. of Beds Daily Occupied. | HOSPITALS. | In-patients. | Out-patient Attendances. | Total Expenditure. | Income. | | | Total Income. | Legacies not included in preceding column. |
|---|---|---|---|---|---|---|---|---|---|---|
| | | | | | | Charitable. | Proprietary. | Patients' Payments. | | |
| | | | | | £ | £ | £ | £ | £ | £ |
| 613 | 529 | Guy's ... ... ... ... | 8,247 | 437,916 | 129,901 | 44,214 | 57,343 | 12,219 | 113,776 | 9,252 |
| 770 | 369 | King's College ... ... ... | 5,032 | 145,170 | 102,499 | 35,073 | 22,627 | 22,910 | 80,610 | 538 |
| 18 | 9 | Phillips' Memorial Homœopathic | 159 | 2,100 | 1,708 | 772 | 504 | 504 | 1,780 | ... |
| 76 | 73 | Miller ... ... ... ... ... | 1,162 | 139,442 | 19,883 | 9,419 | 1,643 | 4,682 | 15,744 | 52 |
| 66 | 52 | St. John's, Lewisham ... ... | 626 | 26,688 | 7,993 | 3,129 | 907 | 3,163 | 7,199 | 100 |
| 632 | 521 | St. Thomas's ... ... ... | 7,687 | 331,278 | 158,353 | 11,555 | 73,042 | 25,984 | 110,581 | 1,373 |
| 306 | 251 | Seamen's ... ... ... ... | 3,429 | 89,660 | 47,053 | 24,651 | 11,008 | 10,839 | 46,498 | 6,204 |
| 60 | 48 | Bolingbroke Hospital ... ... | 701 | 30,922 | 11,367 | 7,132 | 1,327 | 1,368 | 9,827 | ... |
| 32 | 26 | Belgrave, for Children ... ... | 470 | 30,882 | 7,343 | 7,014 | 996 | 367 | 8,377 | 1,200 |
| 76 | 44 | Evelina, for Children ... ... | 609 | 37,663 | 11,113 | 3,456 | 4,742 | 292 | 8,490 | 1,155 |
| 57 | 40 | South Eastern, for Children ... | 500 | 8,580 | 5,072 | 5,111 | 335 | 688 | 6,134 | ... |
| 14 | 11 | British Home for Mothers and Babies ... ... | 206 | 4,608 | 1,851 | 568 | 1,359 | 1,129 | 3,056 | ... |
| 36 | 33 | General Lying-In... ... ... | 958 | 22,781 | 11,680 | 4,439 | 4,032 | 1,530 | 10,001 | ... |
| 50 | 39 | Clapham Maternity & Dispensary | 980 | 5,064 | 4,738 | 1,224 | 1,084 | 4,169 | 6,477 | 200 |
| 80 | 71 | South London, for Women ... | 1,074 | 33,015 | 14,021 | 6,031 | 3,073 | 4,646 | 13,750 | 498 |
| 106 | 81 | Royal Waterloo ... ... ... | 1,035 | 21,880 | 13,338 | 7,694 | 3,463 | 7 | 11,164 | 485 |
| 42 | 33 | Royal Eye ... ... ... ... | 703 | 56,377 | 7,799 | 3,024 | 1,396 | 1,897 | 6,317 | 905 |
| 25 | 14 | Beckenham Cottage ... ... | 330 | 7,116 | 2,838 | 1,916 | 338 | 1,379 | 3,633 | 300 |
| 26 | 19 | Blackheath Cottage ... ... | 229 | 1,297 | 2,688 | 1,402 | 497 | 518 | 2,417 | ... |
| 42 | 15 | Bromley Cottage ... ... ... | 319 | ... | 2,793 | 1,249 | 845 | 584 | 2,678 | ... |
| 22 | 14 | Eltham Cottage ... ... ... | 260 | ... | 2,062 | 1,319 | 214 | 477 | 2,010 | 100 |
| 12 | 6 | Sidcup Cottage ... ... ... | 106 | 203 | 986 | 791 | 118 | 228 | 1,137 | ... |
| 23 | 16 | Victoria Hospital, Kingston ... | 337 | ... | 1,926 | 1,268 | 441 | 340 | 2,049 | 73 |
| 3,184 | 2,314 | | 35,159 | 1,432,642 | 569,005 | 182,451 | 191,334 | 99,920 | 473,705 | 22,435 |
| | | DISPENSARIES. | | | | | | | | |
| ... | ... | Battersea Provident ... ... | ... | 85,000 | 3,214 | 133 | 72 | 3,009 | 3,214 | ... |
| ... | ... | Brixton, &c. ... ... ... | ... | 6,900 | 1,279 | 614 | 888 | 76 | 1,578 | ... |
| ... | ... | Camberwell Provident ... ... | ... | 49,620 | 907 | 249 | 180 | 437 | 866 | ... |
| ... | ... | Clapham ... ... ... ... | ... | 7,766 | 531 | 197 | 165 | 190 | 552 | ... |
| ... | ... | East Dulwich Provident ... | ... | 12,153 | 1,092 | 71 | 56 | 937 | 1,064 | ... |
| ... | ... | Forest Hill Provident ... ... | ... | 18,669 | 573 | 141 | 22 | 399 | 562 | ... |
| ... | ... | Greenwich Provident ... ... | ... | 21,141 | 484 | 46 | 43 | 392 | 481 | ... |
| 3,184 | 2,314 | | 35,159 | 1,633,891 | 577,085 | 183,902 | 192,760 | 105,360 | 482,022 | 22,435 |

### THE MEDICAL CHARITIES OF LONDON.—A SUMMARY OF THE WORK DONE IN 1919.

It will be seen from the following summary that *One hundred and fifty thousand nine hundred and thirty-three* patients were admitted into the Voluntary Hospitals of London during the year 1919, and that the attendances in the Out-Patient Departments and Dispensaries numbered *Six millions and twenty-eight thousand four hundred* at a cost of *£2,477,413*. The Ordinary Income amounted to *£2,006,223*, leaving a deficiency of *£471,190* on the year's work. The Legacies received in 1919 amounted to *£230,694*.

| No. of Beds. | No. of Beds Daily Occupied. | HOSPITALS AND DISPENSARIES. | In-patients. | Out-patient Attendances. | Total Expenditure. | Income. | | | Total Income. | Legacies not included in preceding column. |
|---|---|---|---|---|---|---|---|---|---|---|
| | | | | | | Charitable. | Proprietary. | Patients' Payments. | | |
| | | | | | £ | £ | £ | £ | £ | £ |
| 3,184 | 2,314 | Newington and South District ... | 35,159 | 1,633,891 | 577,085 | 183,902 | 192,760 | 105,360 | 482,022 | 22,435 |
| 1,840 | 1,259 | City and East Central District... | 20,883 | 950,435 | 341,395 | 143,154 | 97,114 | 45,117 | 285,385 | 12,243 |
| 967 | 789 | Westminster District ... ... | 9,303 | 471,331 | 184,911 | 95,942 | 23,120 | 38,057 | 157,119 | 19,993 |
| 2,059 | 1,614 | St. Marylebone, &c., District ... | 19,400 | 659,086 | 371,985 | 175,588 | 59,443 | 68,611 | 303,642 | 52,214 |
| 2,131 | 1,808 | Kensington and West District ... | 19,995 | 688,350 | 359,020 | 130,817 | 57,566 | 76,552 | 264,935 | 99,776 |
| 1,615 | 1,273 | Islington & North-West District | 15,911 | 665,470 | 259,538 | 124,710 | 30,056 | 66,312 | 221,078 | 7,151 |
| 1,947 | 1,535 | Stratford and East-End District | 30,282 | 959,837 | 383,479 | 192,939 | 65,284 | 33,819 | 292,042 | 16,882 |
| 13,743 | 10,592 | | 150,933 | 6,028,400 | 2,477,413 | 1,047,052 | 525,343 | 433,828 | 2,006,223 | 230,694 |

