# A Correction

**Published:** 1920-08-07

**Authors:** C. A. Lampitt

**Affiliations:** Secretary. Memorial Hospital, Mildmay Park.


### THE EDITOR'S LETTER-BOX.

### A Correction.

*To the Editor of* The Hospital.

Sir,—The attention of the Committee of this hospital has been drawn to the statement on page 345 of the issue of The Hospital for June 26 last, in which this hospital is classified in the Stratford and East-End District of London. This is an error and misleading to your readers, and we should be glad if you will be kind enough to correct this in a future issue; the title of the hospital is the "Memorial Hospital," situate in Mildmay Park, which is in the Borough of Islington.—Yours truly, C. A. Lampitt, Secretary.

Memorial Hospital, Mildmay Park.
July 27, 1920.

[The classification is that of the Hospital Sunday Fund authorities, with whom it is suggested Mr. Lampitt should communicate.—Ed. The Hospital.]

### The Dilemma at Gravesend.

*To the Editor of* The Hospital.

